# The role of CD8 PET imaging in guiding cancer immunotherapy

**DOI:** 10.3389/fimmu.2024.1428541

**Published:** 2024-07-12

**Authors:** Jiani Zhang, Bulin Du, Yuxiang Wang, Yan Cui, Shu Wang, Yuxuan Zhao, Yaming Li, Xuena Li

**Affiliations:** Department of Nuclear Medicine, The First Hospital of China Medical University, Shenyang, Liaoning, China

**Keywords:** immunotherapy, CD8 + T cells, PET, clinical application, cancer

## Abstract

Currently, immunotherapy is being widely used for treating cancers. However, the significant heterogeneity in patient responses is a major challenge for its successful application. CD8-positive T cells (CD8^+^ T cells) play a critical role in immunotherapy. Both their infiltration and functional status in tumors contribute to treatment outcomes. Therefore, accurate monitoring of CD8^+^ T cells, a potential biomarker, may improve therapeutic strategy. Positron emission tomography (PET) is an optimal option which can provide molecular imaging with enhanced specificity. This review summarizes the mechanism of action of CD8^+^ T cells in immunotherapy, and highlights the recent advancements in PET-based tracers that can visualize CD8^+^ T cells and discusses their clinical applications to elucidate their potential role in cancer immunotherapy.

## 1 Introduction

Immunotherapy has advanced rapidly in the recent past (1–3). Key methods involving immune checkpoint inhibitors (ICIs) and adoptive cell therapy (ACT) have achieved notable success in the clinical management of various cancers (4, 5). However, durable and effective responses are only observed in a section of patients (6). Efforts have been made to further optimize immunotherapeutic strategies and several studies have attempted to identify potential biomarkers for improving the therapeutic efficacy (7–9).

CD8^+^ T lymphocytes, critical immune effector cells, play a vital role in cancer immunotherapy (10, 11). Studies have shown that improved outcomes correlate positively with CD8^+^ T cell infiltration in several types of tumors (12–14), including melanoma (15), non-small cell lung cancer (NSCLC) (16), breast cancer (17), and cervical cancer (18). In addition to the quantity of infiltration, the functional status of CD8^+^ T cells within the tumor microenvironment (TME), which is influenced by interactions with cells and may change with time, also significantly affect response to therapy (19, 20). Both infiltration and functional status greatly contribute to the heterogeneity in response to immunotherapy (6, 8, 9). Therefore, monitoring of CD8^+^ T cells *in vivo* is crucial for improving patient understanding and implementation of precision medicine.

Tumor biopsy, a conventional invasive method, is used in clinical practice to analyze CD8^+^ T cells. However, due to its inherent limitations, this invasive approach poses several challenges, including difficulty in re-assessment and the inability to provide spatial and dynamic information (21). In contrast, non-invasive methods using various imaging modalities and direct/indirect labeling of target cells or construction of radiolabeled agents play significant roles in monitoring the immune response *in vivo* (22, 23). Positron emission tomography (PET) is a promising molecular technique that can provide whole-body images with considerable specificity and sensitivity (24). The binding of targeted vectors to specific radionuclides forms the foundation of PET radiotracers, and PET enables non-invasive real-time monitoring of the target cells by detecting radionuclide decay emissions (25, 26). PET radiotracers have been extensively used to characterize CD8^+^ T cells, and thus effectively quantify early therapy-induced alterations in immune status (27).

Currently, existing PET radiotracers for visualizing CD8^+^ T cells can be generally divided into two categories: 1) those directly targeting CD8, a dimeric co-receptor, indicating the presence of CD8^+^ cells, and 2) those indirectly reflecting the functionality or status of CD8^+^ T cells by targeting potential biomarkers. This review offers an overview of the development of PET imaging of CD8^+^ T cells, briefly summarizes current information on relevant CD8^+^ T cell biology and innovative PET tracers and discusses the future potential applications of PET in the field of cancer immunotherapy.

## 2 Mechanisms of action of CD8^+^ T cells

### 2.1 CD8^+^ T cells in immunology

Common T cells originate from lymphoid progenitor cells in the red bone marrow. These immature precursor T cells then migrate to the thymus (28). CD8^+^ T cells gradually mature via several specific processes, including the development of the T cell receptor’s (TCR’s) affinity for major histocompatibility complex class-1 (MHC-1), positive selection, and negative selection (28, 29).

The direct interaction between CD8^+^ T cells and corresponding antigens is pivotal for CD8^+^ T cell activation. MHC-1, presented by malignant cells or antigen-presenting cells (APCs), is recognized by the TCR of CD8^+^ T cells (30). Following activation of the TCR signal, additional signals from co-receptors such as CD28 complexed with B7 molecules (CD80/86), along with the influence of cytokines or chemokines, further facilitate the activation of CD8^+^ T cells (31, 32). Consequently, CD8^+^ T cells can identify and target tumor sites. Upon reaching the site, CD8^+^ T cells begin to infiltrate and combat tumor cells.

### 2.2 CD8^+^ T cells in anti-tumor immunity

**Figure 1:**
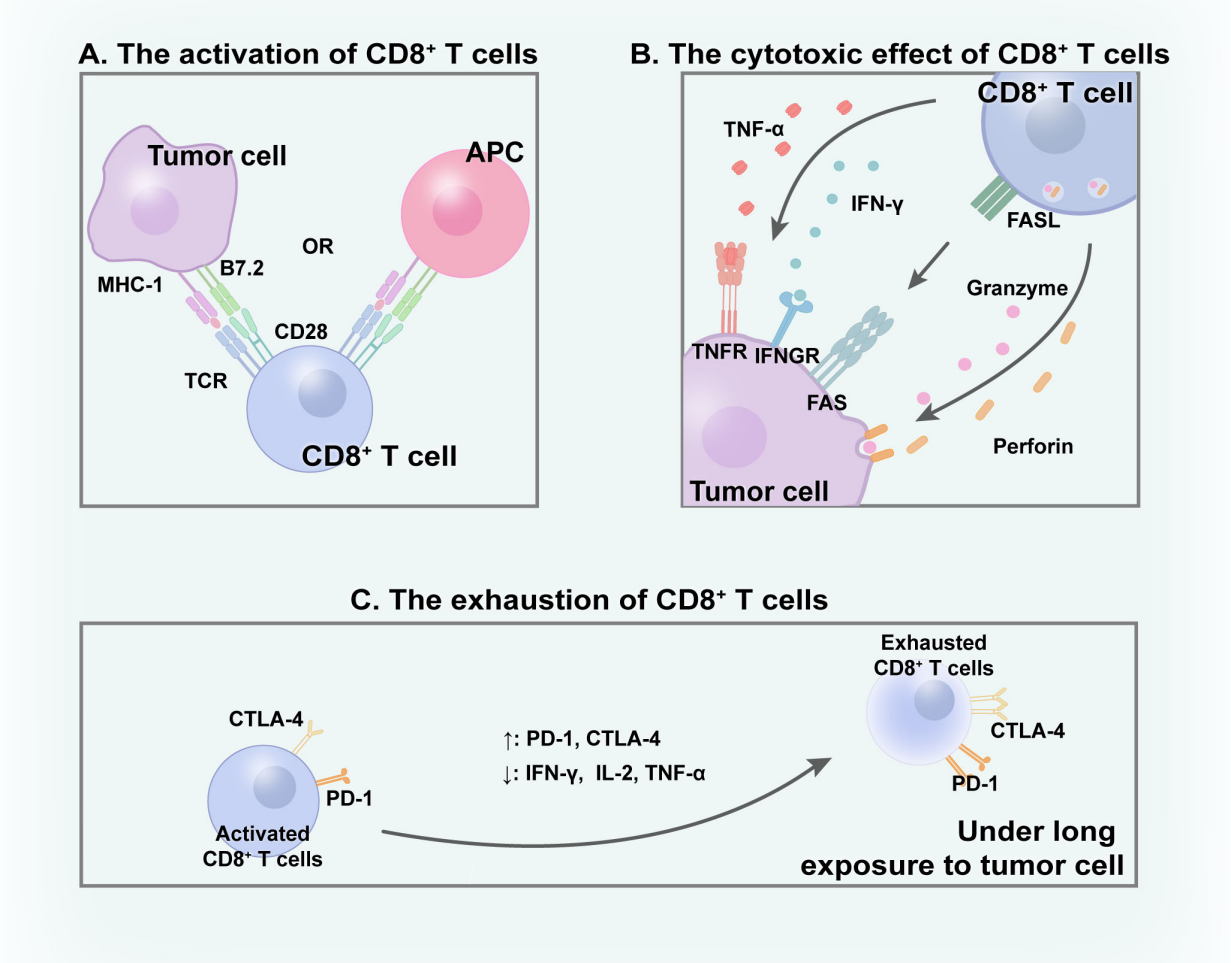
CD8^+^ T cells within TME. **(A)** With the interaction with APC/tumor cells, CD8^+^ T cells get activated and **(B)** subsequently excrete cytotoxic effect to eliminate tumor cells through various mechanisms. **(C)** Under long time exposure to tumor, activated CD8^+^ T cells gradually become exhausted.

Several studies have established the critical function of CD8^+^ T cells in anti-tumor immunity (33, 34). The mechanisms via which CD8^+^ T cells contribute to tumor-killing activity are complex and involve multiple factors. A primary pathway involves the release of granules containing perforin and granzymes by CD8^+^ T cells, directly leading to the apoptosis of malignant cells (34). Perforin creates pores in tumor cell membranes, allowing granzymes to enter the TME and exert cytotoxic effect (35). The FAS ligand (FASL) pathway is another crucial pathway, which is cytotoxic for tumor cells (36). The interaction between FAS on malignant cells and FASL on CD8^+^ T cells triggers a signal that activates the FAS-associated death domain protein, resulting in caspase activation and subsequent apoptosis of tumor cells. Additionally, CD8^+^ T lymphocytes contribute to the destruction of tumor cells by secreting cytokines, including interferon-γ (IFN-γ) and tumor necrosis factor-alpha (37, 38). Various mechanisms collaborate to achieve tumor cell elimination, with several factors playing integral roles, including effector cytokines that impact the CD8^+^ T cells and the dynamic metabolic state of these cells (39, 40) (**Figure 1**).

### 2.3 CD8^+^ T cells in cancer immunotherapy

CD8^+^ T lymphocytes possess the potent ability to kill malignant cells. However, owing to prolonged exposure in the TME, many CD8^+^ T cells gradually exhibit characteristics of “exhaustion” (41, 42). In this state, the proliferation, effector cytokine production, and cytolytic activity of the CD8^+^ T cells tend to decrease, while cell surface expression of inhibitory receptors, including programmed death-1 receptor (PD-1) and cytotoxic T lymphocyte antigen-4 (CTLA-4), increase concurrently (43, 44). Tumor cells exploit this by overexpressing inhibitory immune checkpoints, thereby achieving immune escape and diminishing the effectiveness of the immune response against tumors (45).

**Figure 2:**
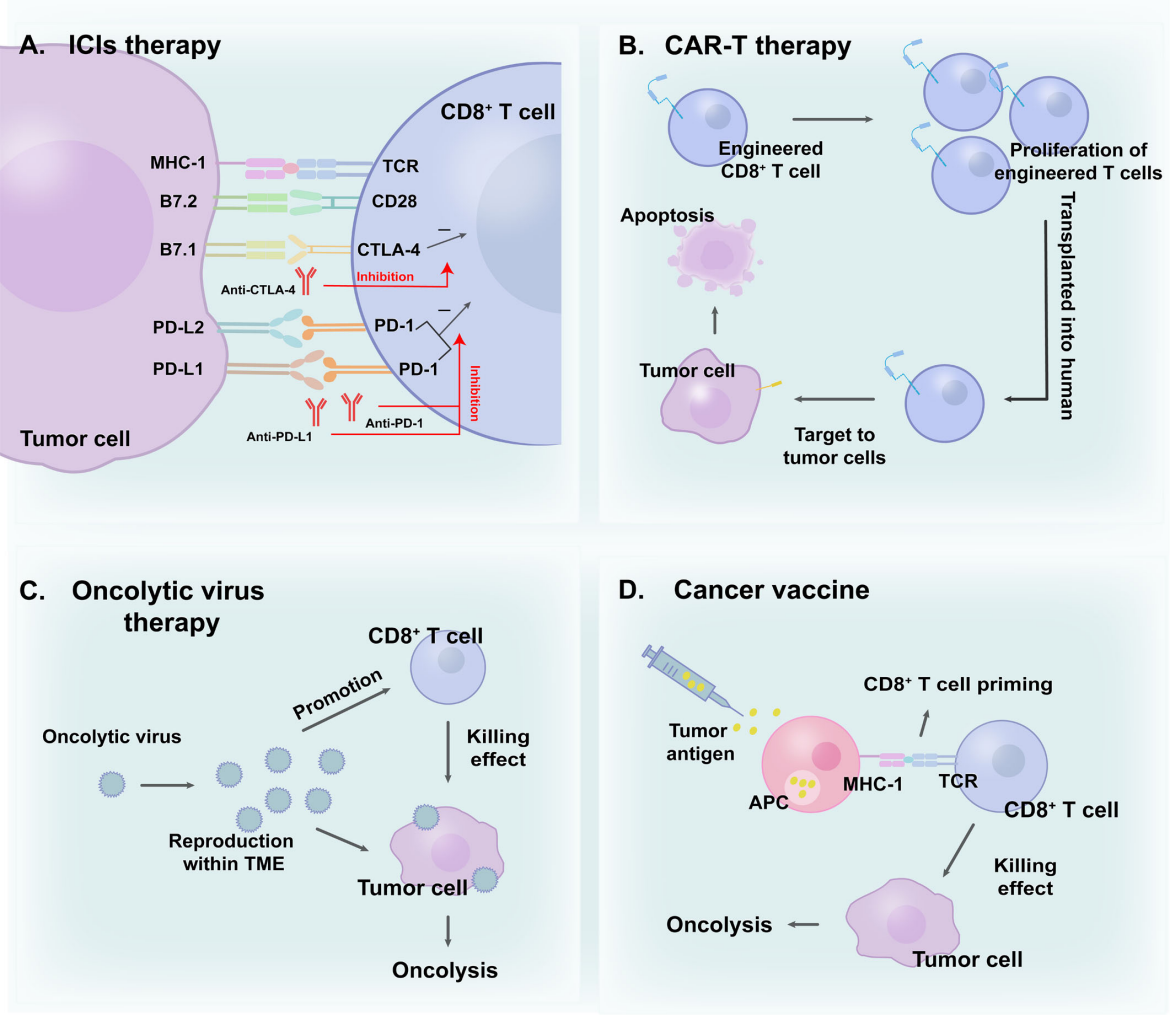
CD8^+^ T cells in **(A)** ICIs therapy; **(B)** CAR-T therapy; **(C)** Oncolytic virus therapy and **(D)** Cancer vaccine. CD8^+^ T cells play a vital role in cancer immunotherapies.

Immunotherapy, which leverages natural immune function to eliminate tumor cells, can be generally categorized into ICIs, ACT, cancer vaccines, oncolytic virus therapies, and cytokine therapies (46). ICIs block immune checkpoint pathways, aiding in the reversal of the exhausted state of CD8^+^ T cells (47, 48). In recent years, ICIs have been shown to improve anti-tumor effects and exhibit excellent results (49, 50). A global study (KEYNOTE-042, NCT02220894) compared first-line monotherapy with pembrolizumab (a representative of ICIs) with platinum-based chemotherapy in patients with locally advanced/metastatic NSCLC without epidermal growth factor receptor/anaplastic lymphoma kinase alterations and programmed death-ligand 1 (PD-L1) tumor proportion score of ≥ 1%. Durable benefit was observed in pembrolizumab groups, in which higher 5-year overall survival (OS) rates were evident (51). Moreover, immunotherapy-based combinations demonstrated promise in further improving outcomes (52). The use of a combination of nivolumab, ipilimumab, and chemotherapy confirmed a significant improvement in OS compared with chemotherapy alone in a phase 3 trial (CheckMate 9LA, NCT03215706) involving patients with NSCLC (53). In addition to ICIs, ACT is a promising option for cancer therapy. Chimeric antigen receptor T cell therapy (CAR-T), based on gene editing in CD8^+^ T cells and reinfusion into the human body, enhances the effectiveness of immune cells (54). These engineered cells can target malignant cells better than other cells and exert cytotoxic effects (55). Moreover, oncolytic virus therapies selectively replicate in tumor cells while promoting the anti-tumor immunity of CD8^+^ T cells (56). Cancer vaccines activate APCs loaded with tumor antigens, inducing efficient CD8^+^ T cell responses (57). To some extent, various immunotherapy methods directly or indirectly boost the anti-tumor effect of CD8^+^ T lymphocytes, aiding in the eradication of tumor cells (58). Furthermore, novel combination therapies with radiotherapy aim to overcome immune resistance and increase CD8^+^ T cell infiltration, thereby enhancing efficacy (59). Consequently, CD8^+^ T lymphocytes play an indispensable part in immunotherapy (**Figure 2**).

## 3 CD8^+^ T cell imaging tracers

PET is a powerful clinical technique (60) utilized for non-invasively and dynamically visualizing CD8^+^ T cells in cancer immunotherapy. Currently, the design and development of tracers for CD8^+^ T cells have improved considerably. Ideal radiotracers should possess high specificity, sensitivity, and a relatively low radiation burden. Both the choice of radionuclides and vectors are critical considerations in this process (27).

Previously, zirconium-89 (^89^Zr) was a popular choice. Owing to its relatively long half-life, this radionuclide pairs well with intact antibodies that also have long serum half-lives, potentially providing reliable information several hours or days after injection (61, 62). Copper-64 (^64^Cu) is another promising candidate for a long-lasting imaging agent that can be used in the human body (63). However, its high signal intensity in the liver and intestines may limit the clinical application of ^64^Cu-labeled probes (27). In contrast, gallium-68 (^68^Ga) and fluorine-18 (^18^F) are better options for obtaining sequential images in clinical settings due to their shorter half-lives (64).

Studies utilizing full-sized antibodies, which are relatively easy to produce as imaging agents, have been successfully used in immuno-PET (27). However, their use is challenging, because their size exceeds the renal filtration cutoff, which may impede diffusion and penetration (65). With the advancement of radiotracers, current choices for targeting vectors extend beyond full-length antibodies, with options such as minibodies, cys-diabodies, and nanobodies emerging. Smaller antibody fragments, particularly nanobodies, which consist solely of a heavy chain structure, are preferred in many studies for their rapid pharmacokinetics, better tissue penetration, and low immunogenicity (66, 67). When combined with radionuclides with short half-lives such as ^68^Ga and ^18^F, nanobodies can facilitate the creation of high-quality images with lower radiation doses, aiding their translation into clinical practice. The currently developed radiotracers used for visualizing CD8^+^ T cells are summarized in the **Table 1**.

**Table 1:** PET tracers visualizing CD8^+^ T cells.

Classification	Target	Radiotracer	Radioisotope	Imaging agent	Study design	Main findings	Author (year)
Quantitative Visualization of CD8^+^ T Cells	CD8	^64^Cu-NOTA-2.43	^64^Cu	Minibody	Preclinical	These two tracers both can detect mouse CD8 expression in preclinical models.	Tavaré et al. (2014) (68)
		^64^Cu-NOTA-YTS169	^64^Cu	Minibody			
		^89^Zr-malDFO-2.43	^89^Zr	Cys-diabody	Preclinical	This tracer can be used to monitor the proliferation, localization of CD8^+^ T cells *in vivo*.	Tavaré et al. (2015) (69)
		^89^Zr-malDFO-169	^89^Zr	Cys-diabody	Preclinical	This tracer is potential to tract endogenous CD8^+^ T cells and evaluate the alterations induced by three distinct immunotherapies (ACT, anti-CD137/4-1BB, anti-PD-L1).	Tavaré et al. (2016) (70)
		^64^Cu-169cDb	^64^Cu	Cys-diabody	Preclinical	This tracer is able to visualize CD8^+^ T cells alterations induced by CpG and αPD-1 therapy.	Seo et al. (2018) (71)
		^89^Zr-DFO-CD8a	^89^Zr	F(ab)’2 fragments	Preclinical	This tracer is of capacity to depict CD8^+^ T cells *in vivo* and predict outcome to a novel PD-1 checkpoint inhibitor (Sym021).	Kristensen et al. (2019) (72)
		^89^Zr-PEGylated anti-CD8 VHH	^89^Zr	Nanobody	Preclinical	This tracer could visualize the increase of CD8^+^ T cells induced by anti-PD-1 treatment and show the potential to evaluate therapy efficacy.	Rashidian et al. (2019) (73)
		^64^Cu-NOTA-CD8a	^64^Cu	F(ab)’2 fragments	Preclinical	This tracer is able to provide images of CD8^+^ T cells changes induced by radiotherapy and CTLA-4 therapy.	Kristensen et al. (2020) (74)
		ZED88082A	^89^Zr	Monovalent antibody	Preclinical	This tracer was developed for assessing CD8^+^ T cells levels *in vivo* without affecting its activity.	Gill et al. (2020) (75)
					Phase 1&2 (NCT04029181)	This uptake of ZED88082A can greatly reflect the dynamic alterations of CD8^+^ T cells in cancer patients undergoing ICIs and shows the prognostic value in the field of immunotherapy.	Kist de Ruijter et al. (2022) (76)
		^89^Zr-Df-IAB22M2C	^89^Zr	Minibody	Phase 1 (NCT03107663)	This tracer passed the safety assessment in human subjects and has the potential to draw a whole-body picture of CD8^+^ T cells in human bodies.	Pandit-Taskar et al. (2020) (77)
					Phase 2 (EudraCT-number 2021-004328-13)	This tracer can assess CD8^+^ T cells infiltration through PET/MRI and show uptake in lymphatic organs.	Schwenck et al. (2023) (78)
		^64^Cu-NOTA-IAB22M2C	^64^Cu	Minibody	Preclinical	This tracer could be used to evaluate CD8^+^ T cells both in peripheral blood and inside GBM.	Nagle et al. (2021) (79)
		^68^Ga-NOTA-SNA006a	^68^Ga	Nanobody	Preclinical	This nanobody-based tracer could rapidly visualize CD8^+^ T cells with great affinity.	Zhao et al. (2021) (80)
		^68^Ga-NODAGA-SNA006	^68^Ga	Nanobody	Phase 1 (NCT05126927)	This novel tracer is able to quantitatively monitor CD8^+^ T cells in human body and dynamically interpret the complex therapy-induced changes.	Wang et al. (2022) (81)
		^18^F-VHH5v2	^18^F	Nanobody	Preclinical	This tracer could detect different CD8^+^ T cells *in vivo* at early time points.	Sriraman et al. (2022) (82)
		^89^Zr-DFO-REGN5054	^89^Zr	Antibody	Preclinical	This tracer could detect CD8^+^ T cells during therapy and is demonstrated safe and well-tolerated in non-human mammals.	Tavaré et al. (2022) (83)
		^89^Zr-IAB42M1-14	^89^Zr	Minibody	Preclinical	This tracer can assess the infiltration of CD8^+^ T cells in tumor, lymphoid tissues and alterations induced by ICOS monotherapy or ICOS/PD-1 combination therapy.	Alsaid et al. (2023) (84)
Functional Visualization of CD8^+^ T Cells	Granzyme B	^68^Ga-NOTA-GZP	^68^Ga	Peptide	Preclinical	The tracer could assess granzyme B expressions reflecting the cytotoxic function and stratify patients.	Larimer et al. (2017) (85)
		^18^F-AlF-mNOTA-GZP	^18^F	Peptide	Preclinical	The uptake of this tracer is greatly associated with the levels of GZB-expressing CD8^+^ T cells and may inform the evaluation of immunotherapy.	Hartimath et al. (2022) (86)
		^68^Ga-grazytracer	^68^Ga	Non-aldehyde peptidomimetics	Phase 1 (NCT05000372)	This tracer is able to distinguish pseudoprogression with tumor progression and is demonstrated the potential to reflect immune response in patients.	Zhou et al. (2022) (87)
	IFN-γ	^89^Zr-anti-IFNγ	^89^Zr	Antibody	Preclinical	This IFN-γ-targeted tracer could detect the alterations of such cytokine and thus indirectly depict the function of immune cells.	Gibson et al. (2018) (88)
		^89^Zr-DFO-NCS-anti-IFN-γ HL-11	^89^Zr	Diabody	Preclinical	This tracer is developed to target IFN-γ *in vivo* and confirmed the one with best properties among distinct choices of linker lengths	Rezazadeh et al. (2022) (89)
	IL-2R	^18^F-FB-IL2	^18^F	Protein	Preclinical	This tracer could specifically attach to IL-2R and possesses the capacity to reveal activated T cells in pathologic conditions.	Di Gialleonardo et al. (2012) (90)
					Phase 1 (NCT02922283)	The feasibility of applying this tracer to human beings was demonstrated safe. Current data shows this tracer can’t evaluate therapy-induced changes.	P.p et al. (2021) (91)
	AraG	^18^F-AraG	^18^F	Small molecule	Preclinical	This tracer could detect the immune response to immunotherapy and has the potential to distinguish patients from responders to non-responders.	Levi et al. (2021) (92)

## 4 Quantitative visualization of CD8^+^ T cells

The immune status within TME is dynamic, with CD8^+^ T cells, other immune cells, and effector molecules being constantly in flux (93, 94). Therefore, real-time monitoring of CD8^+^ T cell infiltration can provide accurate and significant information for personalized therapy. Due to its unique advantages, the PET tracer has emerged as an attractive tool, and many studies have investigated its potential clinical applications.

### 4.1 Feasibility of using CD8 PET tracers

#### 4.1.1 Minibody

CD8-targeted tracers, primarily derived from antibodies, minibodies, cys-diabodies, and nanobodies, have shown promising results. The feasibility of using two ^64^Cu-labeled engineered minibodies, ^64^Cu-NOTA-2.43 and ^64^Cu-NOTA-YTS169, for monitoring CD8^+^ T cells has been confirmed in non-tumor murine models (68). The interaction between murine CD8α (one of the isoforms of CD8) and these tracers was verified without diminishing CD8^+^ T cell populations. Both tracers accumulated in the lymph nodes and spleen in antigen-positive mouse models, with notably decreased uptake in immunodeficient or antigen-depleted models (68). To delineate the TME more accurately in clinical patients, ^64^Cu-DOTA-IAB22M2C, a CD8-targeted minibody, has been effectively used in a xenograft model of orthotopic glioblastoma, demonstrating its ability to monitor both peripheral and intratumoral CD8^+^ T cells (79). Notably, tracer accumulation in the brain indicated that ^64^Cu-DOTA-IAB22M2C could potentially complement ^18^F-FDG, particularly in addressing its limitations in brain tumor imaging.

Building upon these animal studies, clinical trials using tracers with high affinity to human CD8 have been designed. Research on ^89^Zr-Df-IAB22M2C, a ^89^Zr-labeled anti-CD8 minibody, has progressed considerably (77, 95–97). Preclinical studies have demonstrated its uptake in targeted lesions with an ideal target-to-background ratio in mouse models. Subsequently, a phase I first-in-human study (NCT03107663) assessed the optimal mass doses (1.5 mg) and imaging timings (24 hours post-injection) among 15 patients in varying treatment states using ^89^Zr-Df-IAB22M2C (97). The optimal conditions for its clinical application are being investigated in an ongoing phase II clinical trial (NCT03802123).

#### 4.1.2 Nanobody

Existing CD8-targeted minibody-based PET tracers are mainly labeled with ^89^Zr or ^64^Cu which has long half-live. Consequently, these tracers require a long time (usually several hours or days after injection) before the image acquisition and which may cause difficulties for clinical practice. In contrast, nanobodies have emerged as highly potent alternatives, offering rapid targeting, high signal-to-background ratios, and other superior characteristics (98).

Considering the advantages of ^68^Ga over ^89^Zr, a novel CD8-targeted nanobody, ^68^Ga-NOTA-SNA006a, was developed to monitor human CD8 antigen using PET (80). *In vitro* binding assays was conducted to assess the binding capacity of vectors to human CD8 protein and results demonstrated strong binding affinity with positive binding rate constant. *In vivo* studies assessing specificity and stability in humanized mouse models demonstrated its significant uptake in CD8-positive tumors and organs (lung, spleen and liver). An optimized variant with reduced kidney uptake, ^68^Ga-NODAGA-SNA006, was obtained by removing the His6 tag from ^68^Ga-NOTA-SNA006a (81). This nanobody’s ability to quantify CD8^+^ T lymphocytes has been shown not only in preclinical models but also in three lung cancer volunteers (NCT05126927). Notably, a patient who underwent immunotherapy displayed comparably high uptake in tumor lesions, suggesting the potential of this tracer to evaluate therapy-induced changes (81).

### 4.2 Patient stratification for immunotherapy

Immunotherapy has been successfully applied in various clinical settings, particularly in cancer treatment. However, its wider clinical application is hindered by the generally low patient response rate, with immune heterogeneity being a key deterrent. Therefore, visualization of CD8^+^ T cells may be a potent strategy for detecting immune heterogeneity and guiding patient stratification.

Kristensen developed ^89^Zr-DFO-CD8a, which was created from the F(ab’)2 fragments of a rat-anti-mouse CD8a antibody conjugated to the p-SCN-Bn-desferrioxamine chelator (72). This preclinical study investigated the correlation between baseline PET imaging of CD8 levels (tumor-to-heart ratios) and tumor suppression induced by a PD-1 checkpoint inhibitor. Groups with higher numbers of baseline CD8^+^ T lymphocytes exhibited significant tumor suppression, while the efficacy in groups with fewer CD8^+^ T lymphocytes was less pronounced. This finding suggests the potential for stratifying patients from responders to non-responders before starting immunotherapy (72). Notably, significant difference in the maximum ^89^Zr-DFO-CD8a tumor-to-heart ratio between responding and non-responding groups was not observed, highlighting the necessity of understanding the relationship between specific quantitative parameters and the patient population suitable for immunotherapy.

### 4.3 Evaluating the efficacy of immunotherapy

Owing to its unique mechanism, atypical patterns of response may emerge during immunotherapy (99, 100). Pseudoprogression, for example, is an atypical phenomenon that differs from actual progression, characterized by increasing tumor size due to therapy-induced immune cell infiltration (101). The existence of atypical patterns brings great challenges to the evaluation of efficacy in clinical practice (102). CD8 PET tracers can directly monitor the immune alterations caused by immunotherapy unaffected by atypical phenomenon, which may provide accurate evaluation at an early stage and thus favor follow-up decision making.

The use of ^89^Zr-malDFO-169cDb, an ^89^Zr-desferrioxamine-labeled anti-CD8 cys-diabody, enabled tracking of endogenous CD8^+^ T cells and assessment of changes induced by three different immunotherapies in murine models (70). The study compared tumor-to-blood ratios between responders and non-responders in each of the three models, noting that the difference in the anti-PD-L1 therapy group was less significant (70). In addition to monotherapy, Kristensen has evaluated the efficacy of combination therapy. The use of ^64^Cu-NOTA-CD8a for evaluating the response to combination therapy, external radiation therapy with anti-CTLA-4 therapy, has been validated demonstrating the tracer’s utility (74). This finding suggests a potential for using CD8-targeted probes for evaluating therapeutic efficacy and providing valuable information for clinical decision-making.

**Figure 3:**
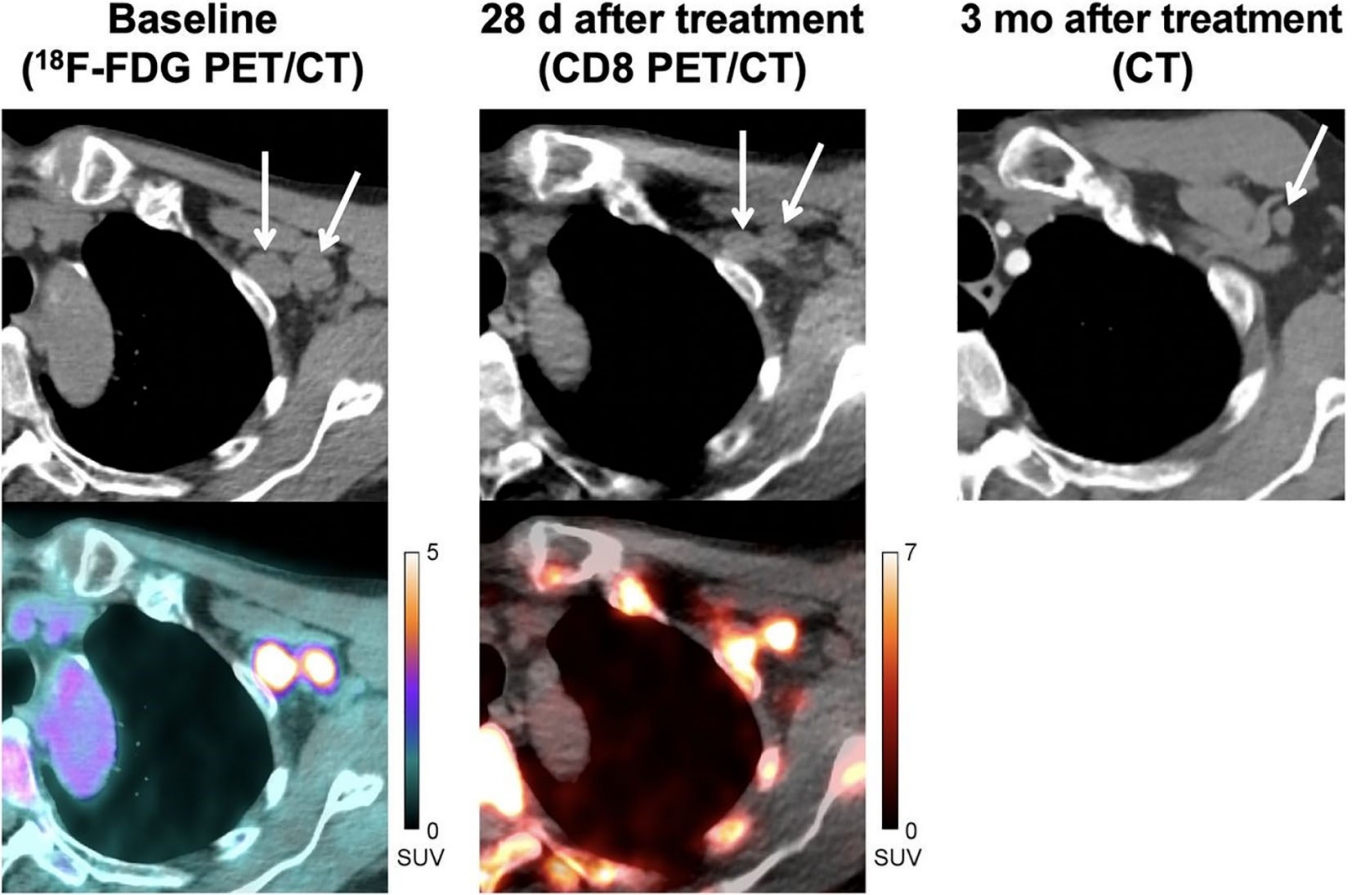
A 71 years old patient (locally advanced stage III melanoma) with immunotherapy (pembrolizumab). CD8 PET/CT images, which performed 28 days after pembrolizumab, show evident uptake in metastases indicating significant infiltration of CD8^+^ T cells. CT images performed 3 months after treatment confirmed the efficacy of immunotherapy. Reproduced from Farwell MD et al. (97).

In clinical settings, a study involving patients with metastatic melanoma undergoing immunotherapy showed that CD8 PET tracer (^89^Zr-Df-IAB22M2C, performed 28 days after immunotherapy) was noticeably incorporated during metastases, indicating infiltration of CD8^+^ T cells. This positive indication was consistent with conventional computed tomography (CT) imaging (performed 3 months after immunotherapy) which suggested a complete response to therapy (**Figure 3**) (97). This study demonstrated the promising potential of CD8 PET in evaluating immunotherapeutic efficacy at rather early stage. Additionally, another clinical study suggested the feasibility of using ^89^Zr-Df-IAB22M2C PET/MRI in assessing CD8^+^ T cells infiltration for efficacy evaluation in a retrospective cohort of eight patients (78). However, future studies involving larger prospective cohorts are required to strengthen these findings.

### 4.4 Dynamic surveillance of ICI therapy

For more effective and precise immunotherapy, dynamic and non-invasive surveillance of the therapeutic process is urgently required. Rashidian used ^89^Zr-PEGylated camelid single-domain antibody fragments to successfully visualize the dynamic increase in CD8^+^ T lymphocytes within tumors during ICI therapy. They observed a significant trend of CD8^+^ T lymphocytes migrating from the periphery to the central position in tumor sites in murine models (73). This finding underscores the unique advantages of dynamic surveillance in this context.

Additionally, the occurrence of adverse events during immunotherapy, which are crucial factors in dynamic surveillance, should not be overlooked. Based on the results of a phase 1/2 trial (NCT04029181), the potential of ZED88082A in detecting inflammation in non-malignant areas has been investigated. The results suggest its ability to characterize CD8-related immune-related adverse events (irAEs) (76). This study investigated the potential applications of CD8-targeted PET tracers and provided practical directions for further clinical translation. However, irAEs may also be induced by other factors, such as B cells, indicating a potential limitation of CD8 PET tracers in obtaining comprehensive information on irAEs. More datasets in the future are required to confirm these findings.

### 4.5 Predicting the prognosis of ICI therapy

**Figure 4:**
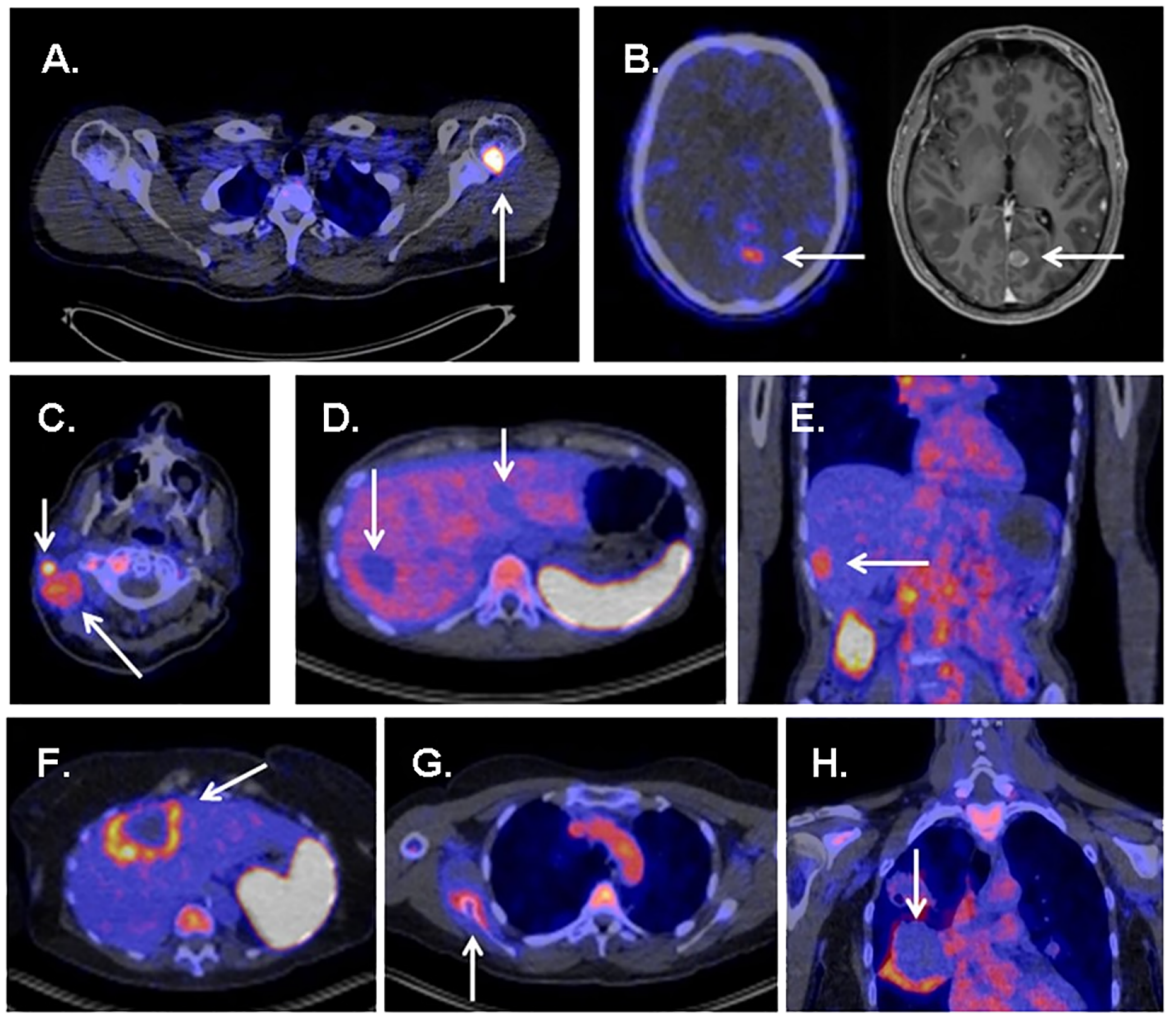
Several examples showing uptake of ^89^ZED88082A in tumor sites and metastases. **(A)** High uptake in bone metastasis of a patient with melanoma. **(B)** Uptake in a brain metastasis of a patient with melanoma. **(C)** Evident uptake in multiple cervical lymph node metastases in a patient with cutaneous squamous cell carcinoma. **(D)** Several liver metastases showing no uptake in a patient with ovarian clear cell carcinoma. **(E)** Uptake in a liver metastasis in a patient with squamous cell oesophageal cancer. **(F)** Liver metastases with rim uptake in a patient with colorectal cancer. (G) Uptake in bone lesion of a patient with squamous cell vulvar cancer. **(H)** Rim uptake in lung metastasis of a patient with cervical cancer. Reproduced from Kist de Ruijter L et al (76).

ZED88082A, based on a monoclonal antibody, was used to visualize CD8 infiltration in patients with solid tumors during ICI therapy (75, 76, 103). Thirty-nine patients (excluding one because of tracer extravasation) were used to assess tracer uptake both at baseline and during therapy (**Figure 4**) (76).

In an average follow-up of 5.6 months, baseline tracer uptake correlated positively with the best overall response per the Response Evaluation Criteria in Solid Tumors. The tracer accumulation in patients with no progressive disease, which included one showing complete response, eight showing partial responses, and four cases of stable disease, was 40% higher than that in patients with progressive disease. The study also found that patients with above-median baseline geometric mean maximum standardized uptake values (SUVmax) (> 5.2) tended to have better progression-free survival (PFS) and superior OS than others (76). This demonstrates the potential of ZED88082A in predicting prognosis for ICI therapy.

Studies focusing on CD8-targeted PET imaging have made significant advancements, and current developments demonstrate the feasibility of using CD8-targeted radiotracers in the field of immunotherapy. To a certain extent, these tracers have improved the efficacy of immunotherapies and may be used to assess the efficacy of newly developed treatments.

## 5 Functional visualization of CD8^+^ T cells

CD8-targeted PET tracers not only reveal the number of effector CD8^+^ T cells but also that of naïve and exhausted CD8^+^ T cells. However, the effector CD8^+^ T cells are the primary subtype contributing to anti-tumor immunity (104). Therefore, targeting of effector CD8^+^ T cells results in a functional representation of all CD8^+^ T cells, offering a comprehensive and accurate assessment of their status after combining both quantitative and functional visualization.

CD8 has been identified to be a reliable target for monitoring immunotherapy in numerous studies. Considering the variable functional states of CD8^+^ T lymphocytes (such as exhausted T cells), and the diverse impacts of various factors on these cells, several researchers have focused on the cytokines produced during the tumor-killing process and throughout T cell activation, which can indicate the activation of cytotoxic T lymphocytes (105).

### 5.1 Granzyme B

**Figure 5:**
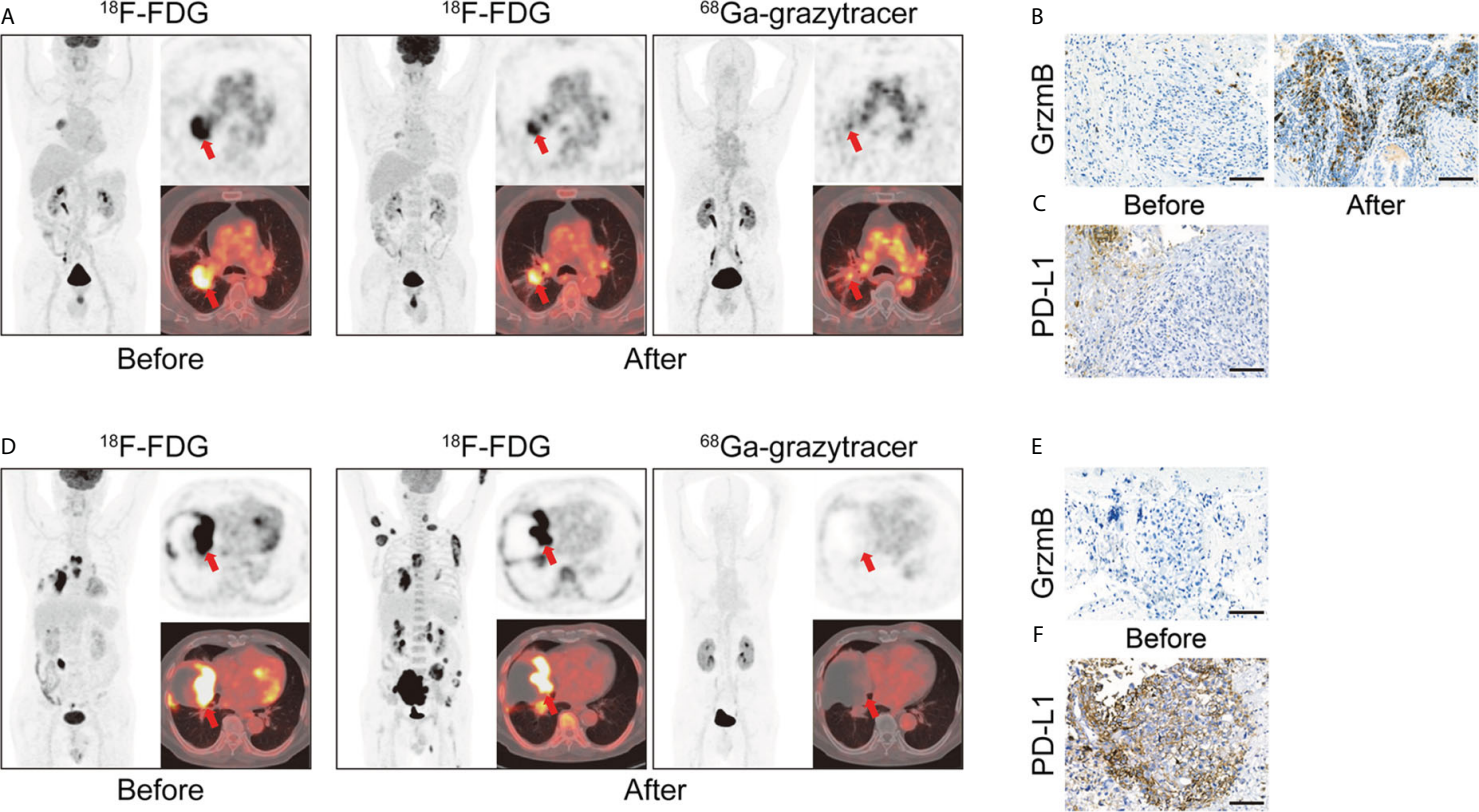
**(A)** A 66 years old patient (lung adenocarcinoma, cT2bN2M0 IIIa) with combination therapy (pemetrexed disodium + cisplatin + toripalimab). ^68^Ga-grazytracer PET/CT images, which performed 3 circles after treatment, demonstrate uptake in tumor lesions indicating the cytotoxic effect against tumor cells (SUVmax 4.1). Follow-up evaluation confirmed a positive prognosis for this patient. **(B, C)** Immunohistochemistry (IHC) staining of granzyme B and PD-L1 of corresponding patient. **(D)** A 70 years old patient (pulmonary sarcomatoid carcinoma, cT4N3M1c IVb) with immunotherapy (pembrolizumab). ^68^Ga-grazytracer PET/CT images, which performed 1 circle after treatment, reveal relatively fewer uptake (SUVmax 2.0) with follow-up evaluation suggesting a negative prognosis for this patient. **(E, F)** IHC staining of granzyme B and PD-L1 of corresponding patient. Reproduced from Zhou H et al. (87).

Granzyme B, released by activated effector CD8^+^ T lymphocytes, participates in the direct tumor-killing mechanism. As a potent representative of the anti-tumor immune response, granzyme B is considered a potential predictive biomarker for cancer therapy (106, 107). Larimer designed a novel peptide-based imaging probe, ^68^Ga-NOTA-GZP, which specifically represents granzyme B expression. They demonstrated a correlation between tracer uptake and therapeutic efficacy, verifying the granzyme B tracer’s potential as an immunotherapy predictive biomarker (85, 108). Another granzyme B-targeted tracer, ^68^Ga-grazytracer, showed comparably higher uptake at tumor sites than ^68^Ga-NOTA-GZP (87). This novel radiotracer, designed by Zhou, has demonstrated the ability to monitor granzyme B levels and the potential to evaluate the efficacy of ICIs and ACT therapy. Furthermore, it can assess intrinsically-induced immune responses, as shown by its ability to distinguish true progression from pseudoprogression in mouse models, potentially complementing the results obtained using ^18^F-FDG. This study also investigated the feasibility of clinical translation in five patients, obtaining positive results consistent with preclinical findings (**Figure 5**) (87).

Evidence suggests that the granzyme B tracer is a valuable tool for characterizing the tumor-killing function in the context of immunotherapy. However, control of the imaging time point remains a hurdle to be overcome due to the variable time window between granzyme B secretion from cytotoxic CD8^+^ T cells and its arrival at malignant cells, which can be influenced by several factors (109).

### 5.2 IFN-γ

IFN-γ, which partially reflects the function of CD8^+^ T cells, may act as a promising biomarker for immuno-PET considering its significant role in the anti-tumor response. Studies have also investigated the feasibility of using IFN-γ as a therapeutic agent in immunotherapy (110, 111). ^89^Zr-anti-IFNγ, developed by Gibson, can be used to assess IFN-γ levels and may be potentially used as a probe for assessing active anti-tumor T cell activity and predicting treatment outcomes in animal models (88). Unlike CD8-targeted tracers, ^89^Zr-anti-IFNγ can directly represent effector function, addressing the limitation that CD8^+^ T cells might become dysfunctional despite the presence of tumor-infiltrating lymphocytes. However, CD8^+^ T cells are not the only subset capable of secreting this cytokine. Depending on the cell subset, IFN-γ might not only exert an anti-tumor effect but also potentially promote tumor progression (112). Therefore, the feasibility and practical value of targeting IFN-γ for immuno-PET has to be verified. Further investigation on IFN-γ-targeted tracers is required not only to evaluate the efficacy of immunotherapy but also to promote the development of IFN-γ-based therapeutic methods.

### 5.3 Arabinofuranosyl guanine

An ^18^F-labeled analog of AraG, which acts as a substrate for deoxyguanosine kinase (dGK), significantly influences T cell activation and functionality, particularly from a metabolic perspective, and can be used to elucidate the status of immune cells (113, 114). ^18^F-AraG can be used to detect the immune response to immunotherapy, as it shows pronounced accumulation in human immune cells (92, 115–116). In contrast, macrophages and dendritic cells negligibly affected the tracer uptake, while a significant increase in ^18^F-AraG uptake correlated with the activation of CD8^+^ T lymphocytes. Unlike ^18^F-FDG, which reflects tumor metabolism, ^18^F-AraG can directly illustrate the course of the immune response, overcoming a major limitation of ^18^F-FDG in immuno-PET applications (92). Levi and colleagues have demonstrated the significant value of ^18^F-AraG in evaluating anti-PD-1 therapy and chemotherapy. However, the variability in individual response kinetics may challenge its clinical translation. Further investigation is required to determine whether this tracer is sufficiently sensitive to detect authentic clinical changes and to optimize its use in the future.

### 5.4 Interleukin-2 receptor

IL-2 plays an active role in both differentiation and activation of T lymphocytes and induces cytotoxic effects by binding to their receptors on target cells (117, 118). IL-2 receptors can be categorized into monomeric, dimeric, and trimeric IL-2Rs. The high-affinity IL-2R consists of CD25, CD122, and CD132 subunits, all of which exist on activated T cells (119). A PET-based tracer, N-(4-18F-fluorobenzoyl) interleukin-2 (^18^F-FB-IL2), can be used to visualize activated T cells in animal models (90, 120). A clinical study (NCT02922283) involving 19 melanoma patients was conducted to investigate the biodistribution and kinetics in human subjects and assess its translational feasibility (91). Furthermore, 11 patients underwent ^18^F-FB-IL2 scans both at baseline and after receiving immunotherapy. Results indicated that the tracer could identify tumor lesions; however, uptake was generally low, and significant correlation was not observed between tracer uptake and therapy-induced changes (91). Therefore, related tracers have to be optimized further to determine whether IL-2R is a suitable target that can accurately and authentically reflect changes induced by ICIs. It is noteworthy that IL-2 has been approved by the Food and Drug Administration of USA for cancer immunotherapy; however, the subsequent results did not meet the initial expectations (121). IL-2R-based PET tracers may be used to develop innovative IL-2 therapeutic strategies.

## 6 Conclusion and future prospects

While immunotherapy is recognized as a groundbreaking advancement in oncology, questions regarding the varying immunogenic statuses of tumors, especially in terms of CD8^+^ T lymphocyte infiltration and their diverse responses to immunotherapy, remain. CD8^+^ T cells play a pivotal role in immune-mediated tumor killing, and hence, CD8-targeted PET radiotracers have become research hotspots. They can be used to track alterations in CD8^+^ T lymphocytes, contributing to the generation of a comprehensive immune profile. A series of preclinical and clinical studies have investigated the potential applications of such radiotracers, demonstrating their capacity to stratify patients, predict outcomes, and evaluate the efficacy of immunotherapies. Notably, baseline CD8 imaging has shown a correlation with better overall responses, indicating its potential as an early predictive biomarker. Furthermore, the emergence of PET tracers that depict the functional status of CD8^+^ T cells, distinguishing activated effector cells, offers the possibility of guiding individualized immunotherapy in a precise manner.

It is noteworthy that the patient numbers and tumor types in most clinical trials are relatively limited. For a more comprehensive investigation on the feasibility of clinical translation and standardized applications, future studies using larger cohorts, multidisciplinary integration, and long-term longitudinal design are required. The current tracers that visualize activated CD8^+^ T cells are limited, and additional biomarkers reflecting the function of CD8^+^ T cells remain underexplored. Some emerging PET imaging parameters require further validation in preclinical studies and multi-center collaborations. A large number of the existing PET tracers based on antibodies have yielded positive results in preclinical studies; however, their radiation burden and long serum half-lives will hinder their subsequent clinical application. Low molecular weight/peptide-based PET tracers coupled with short half-life radioisotopes such as ^68^Ga and ^18^F, which possess the advantages of low radiation burden and shorter image acquisition time, are required for clinical translation. In clinical strategies for tackling malignancies, the trend is shifting toward the use of combination therapy. The dynamic evaluation of such therapies using PET tracers still warrants further investigations.

In conclusion, *in vivo*, systematic, quantifiable, and visual molecular imaging technology may significantly aid clinicians in devising optimal regimens for immunotherapy in precision oncology.

## Author contributions

JZ: Visualization, Writing – original draft, Writing – review & editing. BD: Funding acquisition, Writing – original draft, Writing – review & editing. YW: Writing – original draft, Writing – review & editing. YC: Investigation, Writing – review & editing. SW: Investigation, Writing – review & editing. YZ: Investigation, Writing – review & editing. YL: Funding acquisition, Supervision, Writing – review & editing. XL: Conceptualization, Investigation, Supervision, Writing – review & editing.
